# Percutaneous dilatational tracheotomy in high-risk ICU patients

**DOI:** 10.1186/s13613-021-00906-5

**Published:** 2021-07-28

**Authors:** Enzo Lüsebrink, Alexander Krogmann, Franziska Tietz, Matthias Riebisch, Rainer Okrojek, Friedhelm Peltz, Carsten Skurk, Carsten Hullermann, Jan Sackarnd, Dietmar Wassilowsky, Karl Toischer, Clemens Scherer, Michael Preusch, Christoph Testori, Ulrike Flierl, Sven Peterss, Sabine Hoffmann, Nikolaus Kneidinger, Christian Hagl, Steffen Massberg, Sebastian Zimmer, Peter Luedike, Tienush Rassaf, Holger Thiele, Andreas Schäfer, Martin Orban, the P. D. T. Investigator Group, Stefan Kääb, Stefan Brunner, Mathias Orban, Tobias Petzold, Saliha Kehar, Sara Würbel, Hans-Joachim Stemmler, Leonhard Binzenhöfer, Jan Kleeberger, Antonia Kellnar

**Affiliations:** 1 Intensive Care Unit, Medizinische Klinik und Poliklinik I, Klinikum der Universität München, Marchioninistraße 15, 81377 Munich, Germany; 2 DZHK (German Center for Cardiovascular Research), Partner Site Munich Heart Alliance, Medizinische Klinik und Poliklinik I, Klinikum der Universität München, Munich, Germany; 3 Medizinische Klinik und Poliklinik II, Universitätsklinikum Bonn, Bonn, Germany; 4 Heart Center Leipzig at University of Leipzig, Department of Internal Medicine/Cardiology and Leipzig Heart Institute, Leipzig, Germany; 5 Department of Cardiology and Vascular Medicine, West German Heart and Vascular Center, University Hospital Essen, Essen, Germany; 6 Medizinische Klinik und Poliklinik I, Klinikum Rechts der Isar der Technischen Universität München, Munich, Germany; 7 Klinik Für Kardiologie, Campus Benjamin Franklin, Charité Universitätsmedizin Berlin, Berlin, Germany; 8 Klinik Für Kardiologie I: Koronare Herzkrankheit, Herzinsuffizienz und Angiologie, Universitätsklinikum Münster, Münster, Germany; 9 Klinik Für Anästhesiologie, Klinikum der Universität München, Munich, Germany; 10 Klinik Für Kardiologie, Angiologie und Pneumologie, Herzzentrum Göttingen, Göttingen, Germany; 11 Klinik Für Kardiologie, Angiologie und Pneumologie, Universitätsklinikums Heidelberg, Heidelberg, Germany; 12 Landesklinikum Wiener Neustadt, Wien, Österreich; 13 Herzchirurgische Klinik und Poliklinik, Klinikum der Universität München, Munich, Germany; 14 Institut Für Medizinische Informationsverarbeitung Biometrie und Epidemiologie, Klinikum der Universität München, Munich, Germany; 15 Medizinische Klinik und Poliklinik V, Klinikum der Universität München, Munich, Germany; 16 German Center for Lung Research (DZL), Medizinische Klinik und Poliklinik V, Klinikum der Universität München, Munich, Germany; 17 Klinik Für Kardiologie und Angiologie, Medizinische Hochschule Hannover, Hannover, Germany

**Keywords:** Percutaneous dilatational tracheotomy, Airway management, Anticoagulation, Dual antiplatelet therapy, Bleeding

## Abstract

**Background:**

Percutaneous dilatational tracheotomy (PDT) has become an established procedure in intensive care units (ICU). However, the safety of this method has been under debate given the growing number of critically ill patients with high bleeding risk receiving anticoagulation, dual antiplatelet therapy (DAPT) or even a combination of both, i.e. triple therapy. Therefore, the purpose of this study, including such a high proportion of patients on antithrombotic therapy, was to investigate whether PDT in high-risk ICU patients is associated with elevated procedural complications and to analyse the risk factors for bleeding occurring during and after PDT.

**Methods:**

PDT interventions conducted in ICUs at 12 European sites between January 2016 and October 2019 were retrospectively analysed for procedural complications. For subgroup analyses, patient stratification into clinically relevant risk groups based on anticoagulation and antiplatelet treatment regimens was performed and the predictors of bleeding occurrence were analysed.

**Results:**

In total, 671 patients receiving PDT were included and stratified into four clinically relevant antithrombotic treatment groups: (1) intravenous unfractionated heparin (iUFH, prophylactic dosage) (*n* = 101); (2) iUFH (therapeutic dosage) (*n* = 131); (3) antiplatelet therapy (aspirin and/or P2Y_12_ receptor inhibitor) with iUFH (prophylactic or therapeutic dosage) except for triple therapy (*n* = 290) and (4) triple therapy (DAPT with iUFH in therapeutic dosage) (*n* = 149). Within the whole cohort, 74 (11%) bleedings were reported to be procedure-related. Bleeding occurrence during and after PDT was independently associated with low platelet count (OR 0.73, 95% CI [0.56, 0.92], *p* = 0.009), chronic kidney disease (OR 1.75, 95% CI [1.01, 3.03], *p* = 0.047) and previous stroke (OR 2.13, 95% CI [1.1, 3.97], *p* = 0.02).

**Conclusion:**

In this international, multicenter study bronchoscopy-guided PDT was a safe and low-complication airway management option, even in a cohort of high risk for bleeding on cardiovascular ICUs. Low platelet count, chronic kidney disease and previous stroke were identified as independent risk factors of bleeding during and after PDT but not triple therapy.

**Supplementary Information:**

The online version contains supplementary material available at 10.1186/s13613-021-00906-5.

## Introduction

Percutaneous dilatational tracheotomy (PDT) is an established procedure for medium to long-term ventilation of ICU patients [9, 12, 27]. PDT is mainly indicated in patients with upper respiratory tract obstruction, respirator weaning failure, long-term mechanical ventilation due to neurological disorders and to ensure patency of airways maintaining proper expectoration of bronchial secretion [6, 21, 32]. As a standard of care to prevent long-term translaryngeal intubation complications, PDT has demonstrated its efficacy in preventing laryngeal complications such as mucosal erosion, scarring, stenosis, recurrent laryngeal nerve damage, permanent vocal cord and upper respiratory tract damage [5].

First described by Ciaglia et al., the most common percutaneous dilatational technique allows for procedural application at bedside [4, 20]. Studies have already shown a lower incidence of bleeding and infections associated with PDT in comparison to open surgical tracheotomy [2, 7, 15, 16, 18, 26]. Due to its beneficial safety profile, PDT is widely accepted as a low-complication procedure to ensure a safe patient care [27]. However, particularly at cardiovascular ICUs critically ill patients receiving anticoagulation, dual antiplatelet therapy (DAPT) or even a combination of both, i.e. triple therapy, constitute a significant and steadily growing proportion of patients today. This development is expected to progress, taking into consideration not only the global demographic change with aging population, but also the growing number of invasive cardiovascular interventions with need of intensified and prolonged anticoagulant regimens. Moreover, critically ill patients often suffer from thrombocytopenia and/or coagulation disorders due to sepsis, organ dysfunctions and severity of illness [17]. Several authors suggested that PDT may be considered as relatively contraindicated in patients with coagulation disorders [19, 23] and there are still some relevant questions to answer [9]. Against this background, our international, multicenter study is the largest investigation to date addressing whether PDT can still be regarded as a safe procedure for ICU patients even in a steadily growing cohort with high risk of bleeding and revealing independent risk factors for bleeding during and after PDT.

## Methods

### Study design and patient selection

This retrospective, multicenter cohort study included adult patients (≥ 18 years) undergoing PDT between January 2016 and October 2019. These were hospitalized at cardiovascular ICUs of 12 clinical sites: Klinikum der Ludwig-Maximilians-Universität München (Campus Großhadern), Klinikum der Ludwig-Maximilians-Universität München (Campus Innenstadt), Bonn University Hospital, Klinikum rechts der Isar der Technischen Universität München, Heart center Leipzig at University of Leipzig, Hannover Medical School, Göttingen University Hospital, Essen University Hospital, Heidelberg University Hospital, Münster University Hospital, Landesklinikum Wiener Neustadt and Charité University Clinic Berlin. PDT procedures were indicated due to expected prolonged respirator-dependent ventilation period of seven days and more. All data (i.e. medical history, laboratory analysis, monitoring reports, and clinical notes) were taken from the central clinical database, with subsequent strict data anonymization. The clinical data were collected by one senior clinician from each site. Validity and integrity of the clinical research dataset was controlled by one trained ICU physician and one senior ICU physician and by our statistical team. Standardized definitions for comorbidities and a data dictionary were provided for each clinical site. Patients were excluded from the analysis even if a single value was missing from the dataset. A data analysis and statistical plan was written after the data were assessed. This is the primary analysis of these data which were exclusively collected for present study.

**Fig. 1:**
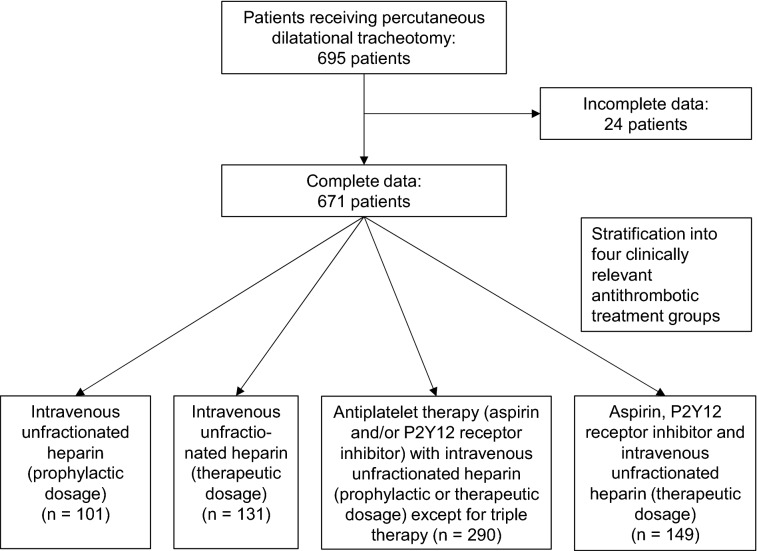
Study flowchart. Flow diagram depicting patient selection

Based on anticoagulation and antiplatelet treatment regimen patients were stratified into four clinically relevant antithrombotic treatment groups with a priori different risks of bleeding: (1) intravenous unfractionated heparin (iUFH) (prophylactic dosage); (2) iUFH (therapeutic dosage); (3) antiplatelet therapy (aspirin (100 mg/day) and/or P2Y_12_ receptor inhibitor, i.e. clopidogrel (75 mg/day), prasugrel (10 mg/day) or ticagrelor (90 mg twice daily)), with iUFH (prophylactic or therapeutic dosage) except for triple therapy (*n* = 290) and (4) triple therapy (DAPT including aspirin (100 mg/day) and a P2Y_12_ receptor inhibitor with iUFH in therapeutic dosage) (Fig. 1). Of note, all ICU patients receiving PDT were treated at least with a prophylactic dose of heparin, thus there was no treatment group without any anticoagulation. In all cases of heparin-induced thrombocytopenia, argatroban was used at equivalent doses. In a second analysis, patients were stratified into 7 antithrombotic subgroups (Additional file 1).

### Percutaneous dilatational technique

**Fig. 2:**
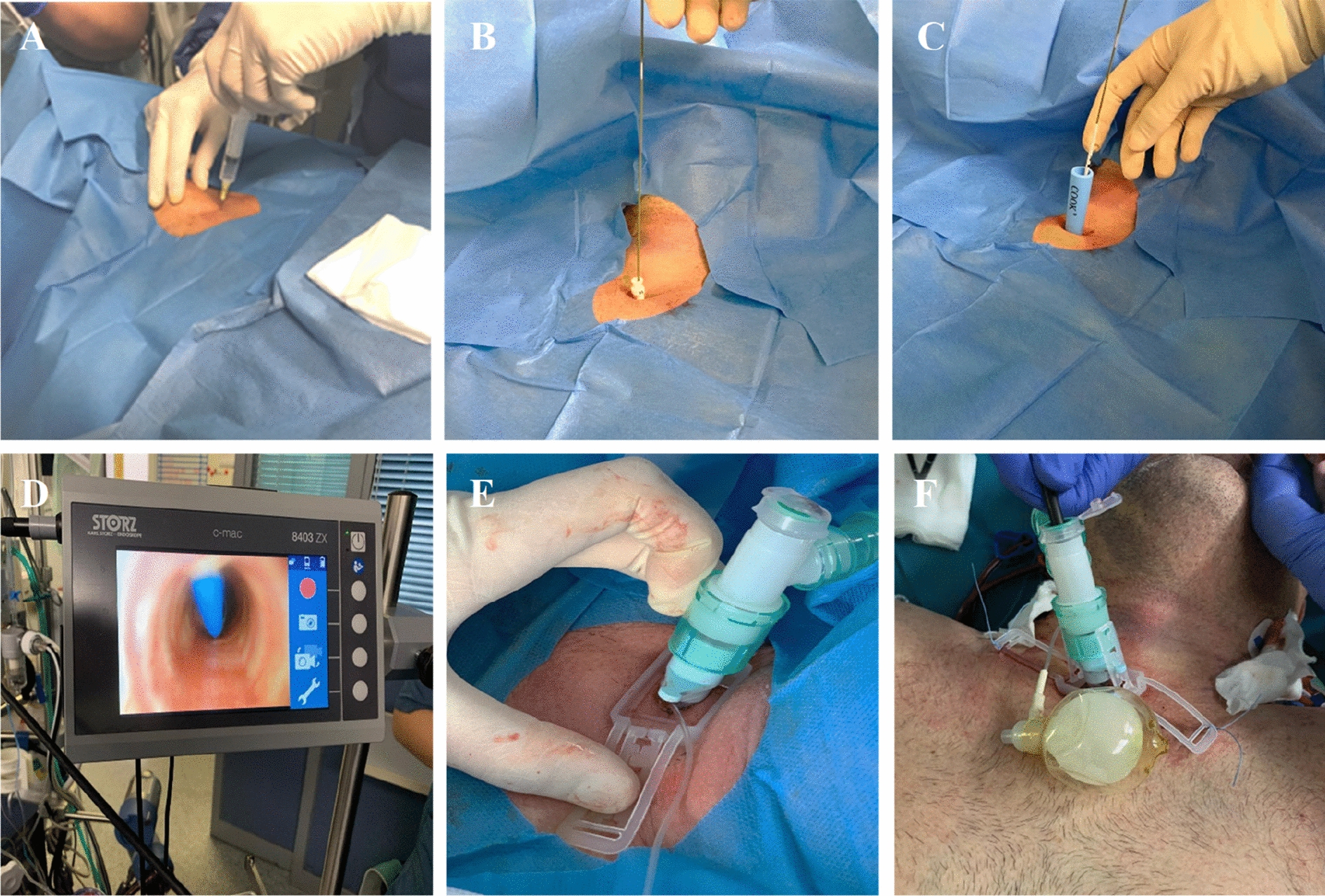
Percutaneous dilatational tracheotomy according to Ciaglia’s technique. Percutaneous dilatational tracheotomy according to Ciaglia’s technique with accompanying bronchoscopy: first, a colour Doppler ultrasound examination may be performed to ensure that no blood vessels run below the intended puncture site. After administration of local anaesthesia (**A**), median puncture of the trachea below the second or third tracheal clasp under bronchoscopic visualization and insertion of a Seldinger wire (**B**) is performed. The Seldinger guidewire is used for bougienage with different dilators (Ciaglia Blue Rhino® Set, Cook Medical) (**C**) under bronchoscopic visualization (**D**). Subsequently, the tracheal cannula is inserted along an introducer and—after bronchoscopy-guided confirmation of correct localization of the cannula within the trachea and subsequent cuff inflation—connected to the respiratory system (**E**). Final bronchoscopic position control of the tracheal cannula (**F**)

PDT was performed according to Ciaglia’s technique with accompanying bronchoscopy as bedside procedure [4, 20] (Fig. 2). Before and throughout PDT, patients were ventilated with FiO_2_ = 1.0 (biphasic positive airway pressure ventilation). At the beginning, an experienced senior ICU consultant performed a bronchoscopy-guided median puncture of the trachea below the second or third tracheal clasp followed by the insertion of a Seldinger wire [11, 14]. In most cases a Doppler ultrasound examination was previously used to ensure that no blood vessels run below the intended puncture site. Dilatation of the tracheal accession side was achieved by bougienage with different dilators increasing in size (Ciaglia Blue Rhino® Set, Cook Medical, Bloomington, USA) along the Seldinger guidewire. Subsequently, the tracheal cannula was inserted by an introducer. Once tracheostomy tube cuff was inflated and correct localization of the tracheal cannula within the trachea was confirmed by bronchoscopy, the tracheal cannula was connected to the respiratory system. Finally, repeated bronchoscopic position control of the tracheal cannula and post-interventional exclusion of pneumothorax by thoracic radiograph were performed. For anaesthesia, all patients received fentanyl (15–50 µg/kg) or sufentanil (5–20 µg/kg), propofol (1.0–2.5 mg/kg KG) and cisatracurium as short-acting muscle relaxant (0.10–0.15 mg/kg). In a timeframe of 4 h pre- and post-procedure, anticoagulation with unfractionated heparin was suspended as standard precautionary measure.

### Ethics approval

The study was conducted in accordance with the Declaration of Helsinki and the institutional and national guidelines and was approved by the local ethics boards (LMU ethics committee reference number: 19–592-KB). The university hospital’s institutional ethic committee waived the need for informed consent because our observational retrospective study did not modify existing diagnostic or therapeutic strategies, clinical data were stored in individual charts on hospital servers, which were biometrically protected, and the extracted data were analysed strictly anonymously.

### Statistical analysis

Statistical analysis was performed using GraphPad Prism® (version 8.0, GraphPad Software Inc.), SPSS® (version 25.0, SPSS Inc.), and R® (version 3.5.2). Kolmogorov–Smirnov test, D’Agostino–Pearson omnibus test, q–q plots and histograms were used to test the normality of data distribution. Normally distributed continuous variables were reported as a mean with standard deviation and non-normally distributed continuous variables as a median with interquartile ranges (25th and 75th percentile). To compare two groups, an independent t-test for normally distributed continuous variables and the Mann–Whitney U test for non-normally distributed continuous variables were used. Categorical variables were reported as absolute numbers and percentages. To compare groups, an exact Fisher’s test was utilized. All tests were 2-tailed, and *p*-values < 0.05 were considered as significant. We used logistic regression models to determine predictors for bleeding during and after PDT. Factors that were suggested to be significant in the univariable analyses, as indicated by *p*-values < 0.2, were included in the multivariable model. No statistical power calculation was conducted prior to the study, and the sample size was based on the available data. Only our statistical team had access to the raw data.

## Results

### Patient baseline characteristics

During the study period, a total of 695 patients, receiving PDT at ICUs of 12 clinical sites, were evaluated. Of these, 24 were excluded due to incomplete data. Patient distribution across the different antithrombotic treatment groups were as follows: (1) iUFH (prophylactic dosage), *n* = 101 (15%); (2) iUFH (therapeutic dosage), *n* = 131 (19%); (3) antiplatelet therapy and/or P2Y12 receptor inhibitor with iUFH (prophylactic or therapeutic dosage) except for triple therapy, *n* = 290 (43%) and (4) triple therapy, *n* = 149 (22%) (Fig. 1). The overall mean age across all treatment groups was 65 ± 14 years, with 66% being male. The majority of patients were admitted at the hospital due to acute myocardial infarction (23%), shock (20%) and acute respiratory failure (18%). PDT was performed 7 to 10 days after primary intubation. Blood analysis at the day of PDT showed haemoglobin (Hb) levels of 9.3 ± 1.5 g/dl, activated partial thromboplastin time (aPTT) values of 43 ± 18 s, mean international normalized ratio (INR) values of 1.2 ± 0.3 and platelet counts of 223 ± 134 g/l. A comprehensive overview of clinically relevant information is shown in Table 1.

**Table 1:** Baseline characteristics of different antithrombotic treatment groups with a total of 671 patients included between January 2016 and October 2019 at ICUs of 12 clinical sites: (1) intravenous unfractionated heparin (iUFH, prophylactic dosage), (2) iUFH (therapeutic dosage), (3) antiplatelet therapy (aspirin (100 mg/day) and/or P2Y12 receptor inhibitor, i.e. clopidogrel (75 mg/day), prasugrel (10 mg/day) or ticagrelor (90 mg twice daily)) with iUFH (prophylactic or therapeutic dosage) except for triple therapy and (4) triple therapy (DAPT including aspirin (100 mg/day) and a P2Y12 receptor inhibitor with iUFH in therapeutic dosage)

Treatment group	(I) Heparin (prophylactic dosage) (*n* = 101)	(II) Heparin (therapeutic dosage) (*n* = 131)	(III) Aspirin and/or P2Y12 receptor inhibitor with Heparin (prophylactic or therapeutic dosage) except for triple therapy (*n* = 290)	(IV) DAPT with heparin (therapeutic dosage) (triple therapy) (*n* = 149)	Overall (*n* = 671)
Age [years] mean ± SD	55 ± 16	64 ± 13	66 ± 12	70 ± 12	65 ± 14
Gender [male] *n* (%)	56 (55)	80 (61)	208 (72)	99 (66)	444 (66)
Body mass index [kg/m^2^] mean ± SD	26.02 ± 5.67	27.87 ± + 5.39	27.16 ± + 5.27	26.99 ± 4.68	27.08 ± 5.25
Reason for hospitalization					
Acute respiratory failure (ARDS, COPD, pneumonia) *n* (%)	38 (38)	32 (24)	43 (15)	6 (4)	119 (18)
Sepsis *n* (%)	12 (12)	11 (8)	9 (3)	7 (5)	39 (6)
Acute myocardial infarciation *n* (%)	0 (0)	0 (0)	78 (27)	77 (52)	155 (23)
Shock (cardiogenic/septic/ hemorrhagic) *n* (%)	28 (28)	34 (26)	48 (17)	23 (15)	133 (20)
Others *n* (%)	23 (23)	54 (41)	112 (39)	36 (24)	225 (34)
Hypertension *n* (%)	49 (49)	78 (60)	212 (73)	116 (78)	455 (68)
Diabetes mellitus *n* (%)	15 (15)	38 (29)	89 (31)	55 (37)	197 (29)
Current smoker *n* (%)	19 (19)	34 (26)	101 (35)	42 (28)	196 (29)
Chronic kidney disease *n* (%)	24 (24)	54 (41)	90 (31)	50 (34)	219 (33)
Previous stroke *n* (%)	5 (5)	14 (11)	42 (14)	21 (14)	82 (12)
Atrial fibrillation *n* (%)	8 (8)	90 (69)	107 (37)	105 (70)	310 (46)
Aspirin *n* (%)	0	0	266 (92)	149 (100)	415 (62%)
P2Y12 inhibitor *n* (%)	0	0	138 (48)	149 (100)	287 (43%)
Creatinine [mg/dl] mean ± SD	1.38 ± 0.87	1.63 ± 0.95	1.60 ± 0.94	1.52 ± 0.85	1.57 ± 0.95
Hemoglobin [g/dl] mean ± SD	9.26 ± 1.55	9.28 ± 1.75	9.28 ± 1.46	9.34 ± 1.33	9.29 ± 1.51
Platelet count [G/l] mean ± SD	216 ± 150	222 ± 131	212 ± 131	249 ± 130	223 ± 134
INR mean ± SD	1.21 ± 0.42	1.23 ± 0.33	1.17 ± 0.30	1.24 ± 0.25	1.20 ± 0.32
aPTT [s] mean ± SD	37.99 ± 15.68	48.65 ± 22.96	39.10 ± 14.15	47.66 ± 16.82	42.68 ± 17.58

All displayed laboratory values were recorded on the day of PDT 4 h prior to procedure. *ARDS* acute respiratory distress syndrome, *COPD* chronic obstructive pulmonary disease, *INR* International Normalized Ratio, *aPTT* activated Partial Thromboplastin Time, *SD* standard deviation

### Antithrombotic treatment group and procedure-related complications

Across all treatment groups, low incidence rates for procedure-related complications were observed (Table 2). Within the whole cohort, 26 (4%) patients were reported to suffer from intraprocedural and 48 (7%) from postprocedural bleeding. In almost all cases haemorrhage was associated with skin bleeding from the entry site and could easily be treated with minimally invasive stitching. None of the bleeding incidents required surgical intervention. In a total of nine cases, persistent subcutaneous bleeding from the entry site necessitated a short-term change to larger tracheal cannula. In two cases there was relevant hypoxia, in one case an intraprocedural venous bleeding caused by the Seldinger guidewire requiring resuscitation. In two other cases, the procedure resulted in resuscitation as a result of anaesthesia-induced hypotension in critically ill patients.

**Table 2:** Procedural related complications during and after PDT differentiated by treatment group

Complication	(I) Heparin (prophylactic dosage) (*n* = 101)	(II) Heparin (therapeutic dosage) (*n* = 131)	(III) Aspirin and/or P2Y12 receptor inhibitor with heparin (prophylactic or therapeutic dosage) except for triple therapy (n = 290)	(IV) DAPT with heparin (therapeutic dosage) (triple therapy) (*n* = 149)	Overall (*n* = 671)	*P*-value
Intraprocedural bleeding *n* (%)	5 (5)	4 (3)	13 (4)	4 (3)	26 (4)	ns
Postprocedural bleeding *n* (%)	9 (9)	9 (7)	19 (7)	11 (7)	48 (7)	ns
Intra-/postproceddural bleeding *n* (%)	14 (14)	13 (10)	32 (11)	15 (10)	74 (11)	ns
Intra-/postproceddural pneumothorax *n* (%)	0 (0)	0 (0)	0 (0)	0 (0)	0 (0)	ns
Intraprocedural accidental cannula dislocation *n* (%)	3 (3)	7 (5)	2 (1)	1 (1)	13 (2)	ns
Intraprocedural accidental tubus dislocation *n* (%)	0 (0)	2 (2)	4 (1)	2 (1)	8 (1)	ns
Postprocedural tracheocutaneous fistula *n* (%)	0 (0)	0 (0)	0 (0)	0 (0)	0 (0)	ns
Postprocedural infection *n* (%)	1 (1)	0 (0)	4 (1)	0 (0)	5 (1)	ns
Postprocedural granulation at the tracheostoma *n* (%)	1 (1)	2 (2)	0 (0)	1 (1)	4 (1)	ns
Postprocedural wound healing *n* (%)	1 (1)	0 (0)	1 (1)	0 (0)	2 (0)	ns
Intraprocedural O_2_ desaturation n (%)	2 (2)	1 (1)	7 (2)	0 (0)	10 (1)	ns
Intraprocedural hypotension *n* (%)	5 (5)	3 (2)	17 (6)	5 (3)	30 (4)	ns
Intraprocedural cardiac arrhythmia *n* (%)	1 (1)	0 (0)	0 (0)	0 (0)	1 (0)	ns
Intraprocedural fracture of tracheal cartilage *n* (%)	1 (1)	9 (7)	13 (4)	6 (4)	29 (4)	ns
Intraprocedural resuscitation *n* (%)	1 (1)	0 (0)	4 (1)	0 (0)	5 (1)	ns
Intra/postprocedural death *n* (%)	0 (0)	0 (0)	0 (0)	0 (0)	0 (0)	ns

*p*-values < 0.05 were considered as significant; *ns* non-significant

Complications related to PDT included tracheal cartilage fracture in 29 (4%) cases. An accidental cannula dislocation occurred in 13 (2%), an accidental endotracheal tube dislocation during PDT procedure in 8 (1%) patients, all of which could be handled through bronchoscopy-guided repositioning or repeated laryngoscopy-guided intubation. An overview of all other PDT-related complications is given in Table 2. Importantly, there were no significant differences between the antithrombotic treatment groups regarding PDT-related complications (Table 2) and interestingly, even high BMI values (> 30 kg/m^2^) were not associated with considerably higher incidence rates of procedural complications (Additional file 1: Table S1).

### Predictors of bleeding during and after PDT

The results of the univariable and multivariable analysis are summarized in Table 3. The multivariable analysis was based on a logistic regression model and revealed low platelet count (OR 0.73, 95% CI [0.56, 0.92], *p* = 0.009), chronic kidney disease (OR 1.75, 95% CI [1.01, 3.03], *p* = 0.047) and previous stroke (OR 2.13, 95% CI [1.1, 3.97], *p* = 0.02) to be independently associated with bleeding during and after PDT. Different antithrombotic treatment regimens including triple therapy were not associated with bleeding risk.

**Table 3:** Predictors of bleeding occurrence during and after PDT

Attribute	Univariate analysis		Multivariate analysis	
	OR [95% CI]	*p*-value	OR [95% CI]	*p*-value
Age (per year)	1 [0.99, 1.02]	0.61		
Gender	0.75 [0.41, 1.32]	0.34		
Body mass index (per kg/m^2^)	1.02 [0.98, 1.07]	0.32		
Hypertension	1.45 [0.84, 2.6]	0.19	1.13 [0.63, 2.09]	0.683
Diabetes mellitus	1.01 [0.58, 1.71]	0.97		
Current smoker	0.56 [0.3, 1.01]	0.07	0.54 [0.28, 0.98]	0.051
Chronic kidney disease	2.2 [1.34, 3.62]	0.002	1.75 [1.01, 3.03]	0.047
Previous Stroke	2.35 [1.24, 4.26]	0.01	2.13 [1.1, 3.97]	0.02
Atrial fibrillation	1.08 [0.66, 1.77]	0.76		
Heparin (prophylactic dosage)	0.99 [0.46, 1.93]	0.99		
Heparin (therapeutic dosage)	0.92 [0.47, 1.68]	0.79		
Aspirin and heparin (prophylactic dosage)	0.94 [0.27, 2.43]	0.9		
Aspirin and heparin (therapeutic dosage)	0.86 [0.4, 1.67]	0.68		
P2Y12 receptor and heparin (therapeutic dosage)	1.22 [0.28, 3.65]	0.76		
DAPT with heparin (prophylactic dosage)	1.11 [0.56, 2.04]	0.75		
Triple-therapy	0.93 [0.49, 1.66]	0.82		
Creatinine (per mg/dl)	1.23 [0.97, 1.55]	0.08	1.06 [0.79, 1.38]	0.698
Hemoglobin (per g/dl)	0.92 [0.77, 1.08]	0.32		
Platelet count (per G/l)	0.7 [0.55, 0.88]	0.003	0.73 [0.56, 0.92]	0.009
INR	1.59 [0.8, 2.85]	0.14	0.61 [0.13, 1.75]	0.438
aPTT (per s)	1.01 [0.99, 1.02]	0.32		

Logistic regression models were used to determine important predictors for bleeding during and after PDT. Factors with a *P*-value < 0.2 in the univariable analyses were included in the multivariable model. *P*-values < 0.05 were considered significant. *OR* Odds ratio, *95% CI* 95% confidence interval, *INR* International Normalized Ratio, *aPTT* activated Partial Thromboplastin Time

## Discussion

In the current study, we provide strong evidence to revoke the perception of intensified antithrombotic therapy as contraindication for PDT. Despite the inherently high risk of bleeding in this cohort, PDT could be performed efficiently, and procedure-related complication rate was very low at all. This is a remarkable finding, also in light of 149 (22%) patients undergoing PDT even on triple therapy. Overall, no case of death was directly attributed to PDT procedure and there were only 5 (0.7%) procedure-related resuscitations, all of which could be ended with ROSC and without permanent patient damage. Considering the findings of this study, we conclude that potential complications of long-term translaryngeal intubation outweigh the low PDT-related complication rate. Interestingly, clinical practice showed that mild bleeding at the tracheal cannula incision site can be easily controlled or prevented with a minimally invasive suture technique consisting of two single-head sutures at the incision point next to the tracheal cannula [22]. For this reason, this technique may be considered to be routinely used in all PDTs. Importantly, bronchoscopy guidance, combined with optional pre-procedural ultrasound, should be routinely performed to better determine the exact needle insertion site and tracheostomy positioning to increase safety during the procedure [9].

The present international, multicenter study provides the most comprehensive safety evaluation of current PDT practice in the largest cohort of high bleeding risk patients on antithrombotic therapy. The results further underline the superiority of PDT in procedural safety—in particular also regarding bleeding complications—as compared with surgical tracheotomy [1, 11, 16, 25]. Based on present investigation, PDT, if correctly indicated and performed, can be recommended as a safe method of securing airway, even in a cohort with high risk of bleeding resulting from anticoagulation, DAPT or a combination of both. Despite their inherent risk to provoke hemorrhage compared to physiologic hemostasis, no antithrombotic treatment regimen considered in our study was an independent risk factors for bleeding complications. Current proof of safety is accompanied by many well documented complementing benefits of PDT such as improvement of patient comfort, oral hygiene and nurse care, reduced need for sedation and analgesia, spontaneous closure of the wound after decannulation and barely visible scar, reduced work of breathing, shortened weaning period from the respirator as well as overall shortened ICU stay [3, 8, 10, 13, 24, 28]. Of note, PDT was performed 7 to 10 days after primary intubation. Trials comparing earlier and later tracheostomy found no difference in hospital length of stay, ICU length of stay and overall mortality and meta-analyses showed that timing was not associated with all-cause ICU mortality or 1-year mortality [30, 31, 33]. Nevertheless, the question of the optimal time for tracheostomy still remains unanswered and further prospective randomized trials are needed to answer this question [9].

However, a systematic registration of independent risk factors for bleeding complications, namely chronic kidney disease, previous stroke and platelet count, could enable further improvement of PDT procedure and outcome. Notably, this is the first time that these risk factors have been identified. Consequent optimization of the attributes mentioned above, such as timely correction of low platelet counts under consideration of contraindications of platelet transfusion and its associated risk of thrombosis, may contribute to a further reduction of bleeding risk. Additionally, in order to identify patients with coagulation disorders, including platelet dysfunctions, the establishment of extended platelet function and coagulation testing, i.e. ADP-induced platelet aggregation [29], may be discussed before performing PDT procedure. Furthermore, mentioned predictors allow better identification of patients with an a priori increased risk of bleeding, for whom close monitoring with optimal awareness and readiness for complication management is required.

An additional interest of this study was to evaluate the role of highly prevalent obesity for PDT-related complications. In early investigations, obesity (BMI ≥ 30) increased the procedure-related complication rate up to 15%, comparing to 8% in patient with BMI < 30 [21]. Contrary to that, we did not find a significant increase in PDT-related complications in patients with high BMI values in accordance with more recent studies [27]. Consequently, the findings from our investigation did not regard obesity as general contraindication for performing PDT, although anatomical challenges caused by an increased amount of pre-tracheal tissue for example as well as derangements that may occur during airway management should be considered thoroughly in this patient group to minimize the risk of procedure-related complications as previously described [27].

## Limitations

The limited sample size of events reduces the reproducibility and reliability of the observations. Furthermore, due to the retrospective nature of the present investigation, there are limitations inherent to observational studies resulting mainly from a lack of randomization and blinding, namely unmeasured confounders, selection bias, incomplete datasets, and the lack of strict protocols covering all aspects of care. With a decentralized international, multicenter approach, we aimed to limit the potential influence of single centre bias.

## Conclusions

The present study, based on the analysis of 671 ICU patients, showed that bronchoscopy-guided PDT is a safe procedure for airway management in ICU patients, even in the subpopulation of patients treated with dual antiplatelet therapy and therapeutic anticoagulation (triple therapy). Chronic kidney disease, previous stroke and low platelet count were independent risk factors for PDT-related bleeding complications.

## Supplementary Information

**Additional file 1: Table S1.** Complications during and after PDT differentiated by body mass index (BMI). *p*-values < 0.05 were considered as significant; ns: non-significant. Of note, there were 9 patients with BMI value < 16 kg/m^2^ and 12 patients with BMI value > 40 kg/m^2^ who were not included. **Table S2.** Baseline characteristics (based on seven treatment groups): I) Intravenous unfractionated heparin (iUFH) (prophylactic dosage), II) iUFH (therapeutic dosage), III) aspirin (100 mg/day) and iUFH (prophylactic dosage) IV) aspirin (100 mg/day) and iUFH (therapeutic dosage), V) P2Y12 receptor inhibitor, i.e. clopidogrel (75 mg/day), prasugrel (10 mg/day) or ticagrelor (90 mg twice daily) with iUFH (therapeutic dosage), VI) DAPT including aspirin (100 mg/day) and a P2Y12 receptor inhibitor with iUFH (prophylactic dosage) and VII) DAPT with iUFH (therapeutic dosage), i.e. triple therapy. All displayed laboratory values were recorded on the day of PDT 4 h prior to procedure. ARDS, acute respiratory distress syndrome; COPD, chronic obstructive pulmonary disease; INR, International Normalized Ratio; aPTT, activated Partial Thromboplastin Time; SD, standard deviation. **Table S3.** Procedural related complications during and after PDT (based on seven treatment groups). *p*-values < 0.05 were considered as significant; ns: non-significant.

## Data Availability

The datasets analysed during the current study are available from the corresponding author on reasonable request. The data are not publicly available due to ethical restrictions and legal constraints. Readers may contact Dr Orban for reasonable requests for the data. De-identified data may be provided after approval from the ethical review board.
