# Supplementary material for: Percutaneous dilatational tracheotomy in high-risk ICU patients
Source: Ann Intensive Care. 2021 Jul 28;11:116. doi: 10.1186/s13613-021-00906-5 (PMC8319261; doi:10.1186/s13613-021-00906-5)
Supplement: Supplementary file 1 — Additional file 1: Table S1. Complications during and after PDT differentiated by body mass index (BMI). p-values < 0.05 were considered as significant; ns: non-significant. Of note, there were 9 patients with BMI value < 16 kg/m2 and 12 patients with BMI value > 40 kg/m2 who were not included. Table S2. Baseline characteristics (based on seven treatment groups): I) Intravenous unfractionated heparin (iUFH) (prophylactic dosage), II) iUFH (therapeutic dosage), III) aspirin (100 mg/day) and iUFH (prophylactic dosage) IV) aspirin (100 mg/day) and iUFH (therapeutic dosage), V) P2Y12 receptor inhibitor, i.e. clopidogrel (75 mg/day), prasugrel (10 mg/day) or ticagrelor (90 mg twice daily) with iUFH (therapeutic dosage), VI) DAPT including aspirin (100 mg/day) and a P2Y12 receptor inhibitor with iUFH (prophylactic dosage) and VII) DAPT with iUFH (therapeutic dosage), i.e. triple therapy. All displayed laboratory values were recorded on the day of PDT 4 h prior to procedure. ARDS, acute respiratory distress syndrome; COPD, chronic obstructive pulmonary disease; INR, International Normalized Ratio; aPTT, activated Partial Thromboplastin Time; SD, standard deviation. Table S3. Procedural related complications during and after PDT (based on seven treatment groups). p-values < 0.05 were considered as significant; ns: non-significant. [file 13613_2021_906_MOESM1_ESM.docx]

**- Additional file -**

**Procedure related complications according to body mass index**

| **Body mass index [kg/m²]** | **16-19  (n = 31)** | **20-24  (n = 195)** | **25-30  (n = 294)** | **31-40  (n = 130)** | **P-value** |
| --- | --- | --- | --- | --- | --- |
| Intraprocedural bleeding n (%) | 1 (3) | 5 (3) | 11 (4) | 9 (7) | ns. |
| Postprocedural bleeding n (%) | 1 (3) | 14 (7) | 20 (7) | 11 (8) | ns. |
| Pneumothorax n (%) | 0 (0) | 0 (0) | 0 (0) | 0 (0) | ns. |
| Accidental cannula dislocation n (%) | 0 (0) | 3 (1) | 4 (1) | 3 (2) | ns. |
| Accidental tubus dislocation n (%) | 0 (0) | 3 (2) | 3 (1) | 1 (1) | ns. |
| Tracheocutaneous fistula n (%) | 0 (0) | 0 | 0 (0) | 0 (0) | ns. |
| Infection n (%) | 0 (0) | 4 (2) | 1 (0) | 0 (0) | ns. |
| Granulation at the tracheostoma n (%) | 0 (0) | 2 (1) | 2 (1) | 0 (0) | ns. |
| Wound healing n (%) | 0 (0) | 2 (1) | 0 (0) | 0 (0) | ns. |
| O_2_ desaturation n (%) | 0 (0) | 4 (2) | 4 (1) | 2 (2) | ns. |
| Hypotension n (%) | 0 (0) | 12 (6) | 14 (5) | 4 (3) | ns. |
| Cardiac arrhytmia n (%) | 0 (0) | 0 (0) | 1 (0) | 0 (0) | ns. |
| Fracture of tracheal cartilage n (%) | 2 (6) | 7 (4) | 12 (4) | 6 (5) | ns. |
| Resuscitation n (%) | 0 (0) | 1 (1) | 3 (1) | 1 (1) | ns. |
| Death n (%) | 0 (0) | 0 (0) | 0 (0) | 0 (0) | ns. |

**Table S1.** Complications during and after PDT differentiated by body mass index (BMI). p-values < 0.05 were considered as significant; ns: non-significant. Of note, there were 9 patients with BMI value < 16 kg/m^2^ and 12 patients with BMI value > 40 kg/m^2^ who were not included.

**Baseline characteristics (based on seven treatment groups)**

| **Treatment group** | | **Heparin (prophylactic dosage) (I)**  **(n = 101)** | **Heparin (therapeutic dosage) (II)**  **(n = 131)** | **Aspirin and heparin (prophylactic dosage) (III)**  **(n = 46)** | **Aspirin and heparin**  **(therapeutic dosage) (IV)**  **(n = 106)** | **P2Y12 receptor**  **and heparin (therapeutic dosage) (V)**  **(n = 24)** | **DAPT with heparin (prophylactic dosage) (VI)**  **(n = 114)** | **DAPT with heparin (therapeutic dosage) (triple therapy) (VII)**  **(n = 149)** | **Overall**  **(n = 671)** |
| --- | --- | --- | --- | --- | --- | --- | --- | --- | --- |
| Age [years] mean ± SD | | 55±16 | 64±13 | 65±10 | 66±12 | 75±11 | 65±12 | 70±12 | 65±14 |
| Gender [male] n (%) | | 56 (55) | 80 (61) | 33 (72) | 70 (66) | 18 (75) | 87 (76) | 99 (66) | 444 (66) |
| Body mass index [kg/m²] mean ± SD | | 26.02±5.67 | 27.87±+5.39 | 27.18±5.18 | 27.36±5.74 | 27.73±5.85 | 26.84±4.77 | 26.99±4.68 | 27.08±5.25 |
| Reason for hospitalization | Acute respiratory failure (ARDS, COPD, Pneumonia)  n (%) | 38 (38) | 32 (24) | 18 (39) | 24 (23) | 1 (5) | 0 (0) | 6 (4) | 119 (18) |
|  | Sepsis n (%) | 12 (12) | 11 (8) | 2 (4) | 6 (6) | 1 (5) | 0 (0) | 7 (5) | 39 (6) |
|  | Acute myocardial infarciation  n (%) | 0 (0) | 0 (0) | 1 (2) | 0 (0) | 3 (13) | 74 (65) | 77 (52) | 155 (23) |
|  | Shock (cardiogenic/septic/ hemorrhagic) n (%) | 28 (28) | 34 (26) | 7 (15) | 14 (13) | 8 (33) | 19 (17) | 23 (15) | 133 (20) |
|  | Others n (%) | 23 (23) | 54 (41) | 18 (39) | 62 (58) | 11 (46) | 21 (18) | 36 (24) | 225 (34) |
| Hypertension n (%) | | 49 (49) | 78 (60) | 34 (74) | 74 (70) | 24 (100) | 80 (70) | 116 (78) | 455 (68) |
| Diabetes mellitus n (%) | | 15 (15) | 38 (29) | 14 (30) | 36 (34) | 12 (50) | 27 (24) | 55 (37) | 197 (29) |
| Current smoker n (%) | | 19 (19) | 34 (26) | 15 (33) | 19 (18) | 7 (29) | 60 (53) | 42 (28) | 196 (29) |
| Chronic kidney disease n (%) | | 24 (24) | 54 (41) | 11 (24) | 34 (32) | 14 (58) | 31 (27) | 50 (34) | 219 (33) |
| Previous Stroke n (%) | | 5 (5) | 14 (11) | 9 (20) | 17 (16) | 3 (13) | 13 (11) | 21 (14) | 82 (12) |
| Atrial fibrillation n (%) | | 8 (8) | 90 (69) | 7 (15) | 70 (66) | 21 (88) | 9 (8) | 105 (70) | 310 (46) |
| Aspirin | | 0 | 0 | 46 | 106 | 0 | 114 | 149 | 415 |
| P2Y12 inhibitor | | 0 | 0 | 0 | 0 | 24 | 114 | 149 | 287 |
| Creatinine [mg/dl] mean ± SD | | 1.38±0.87 | 1.63±0.95 | 1.45±0.83 | 1.68±0.96 | 1.85±0.96 | 1.54±0.94 | 1.52±0.85 | 1.57±0.95 |
| Hemoglobin [g/dl] mean ± SD | | 9.26±1.55 | 9.28±1.75 | 9.24±1.66 | 9.34±1.39 | 8.40±0.87 | 9.43±1.49 | 9.34±1.33 | 9.29±1.51 |
| Platelet count [G/l] mean ± SD | | 216±150 | 222±131 | 238±164 | 192±111 | 175±98 | 228±135 | 249±130 | 223±134 |
| INR mean ± SD | | 1.21±0.42 | 1.23±0.33 | 1.12±0.17 | 1.19±0.27 | 1.31±0.71 | 1.14±0.20 | 1.24±0.25 | 1.20±0.32 |
| aPTT [sec] mean ± SD | | 37.99±15.68 | 48.645±22.96 | 35.50±10.72 | 44.16±17.70 | 43.48±12.92 | 34.93±9.51 | 47.66±16.82 | 42.68±17.58 |

**Table S2.** Baseline characteristics (based on seven treatment groups): I) Intravenous unfractionated heparin (iUFH) (prophylactic dosage), II) iUFH (therapeutic dosage), III) aspirin (100 mg/day) and iUFH (prophylactic dosage) IV) aspirin (100 mg/day) and iUFH (therapeutic dosage), V) P2Y12 receptor inhibitor, i.e. clopidogrel (75 mg/day), prasugrel (10 mg/day) or ticagrelor (90 mg twice daily) with iUFH (therapeutic dosage), VI) DAPT including aspirin (100 mg/day) and a P2Y12 receptor inhibitor with iUFH (prophylactic dosage) and VII) DAPT with iUFH (therapeutic dosage), i.e. triple therapy. All displayed laboratory values were recorded on the day of PDT 4 hours prior to procedure. ARDS, acute respiratory distress syndrome; COPD, chronic obstructive pulmonary disease; INR, International Normalized Ratio; aPTT, activated Partial Thromboplastin Time; SD, standard deviation.

**Procedure related complications (based on seven treatment groups)**

| **Complication** | **Heparin (prophylactic dosage) (I)**  **(n = 101)** | **Heparin (therapeutic dosage) (II)**  **(n = 131)** | **Aspirin and heparin (prophylactic dosage) (III)**  **(n = 46)** | **Aspirin and heparin**  **(therapeutic dosage) (IV)**  **(n = 106)** | **P2Y12 receptor**  **and heparin (therapeutic dosage) (V)**  **(n = 24)** | **DAPT with heparin (prophylactic dosage) (VI)**  **(n = 114)** | **DAPT with heparin (therapeutic dosage) (triple therapy) (VII)**  **(n = 149)** | **Overall  (n = 671)** | **P-value** |
| --- | --- | --- | --- | --- | --- | --- | --- | --- | --- |
| Intraprocedural bleeding n (%) | 5 (5) | 4 (3) | 0 (0) | 3 (3) | 3 (13) | 7 (6) | 4 (3) | 26 (4) | ns. |
| Postprocedural bleeding n (%) | 9 (9) | 9 (7) | 5 (11) | 8 (8) | 0 (0) | 6 (5) | 11 (7) | 48 (7) | ns. |
| Intra-/postproceddural  bleeding n (%) | 14 (14) | 13 (10) | 5 (11) | 11 (11) | 3 (13) | 13 (11) | 15 (10) | 74 (11) | ns. |
| Intra-/postproceddural  Pneumothorax n (%) | 0 (0) | 0 (0) | 0 (0) | 0 (0) | 0 (0) | 0 (0) | 0 (0) | 0 (0) | ns. |
| Intraprocedural accidental cannula dislocation n (%) | 3 (3) | 7 (5) | 0 (0) | 2 (2) | 0 (0) | 0 (0) | 1 (1) | 13 (2) | ns. |
| Intraprocedural accidental tubus dislocation n (%) | 0 (0) | 2 (2) | 0 (0) | 1 (1) | 0 (0) | 3 (3) | 2 (1) | 8 (1) | ns. |
| Postprocedural tracheocutaneous fistula n (%) | 0 (0) | 0 (0) | 0 (0) | 0 (0) | 0 (0) | 0 (0) | 0 (0) | 0 (0) | ns. |
| Postprocedural infection n (%) | 1 (1) | 0 (0) | 0 (0) | 0 (0) | 1 (4) | 3 (3) | 0 (0) | 5 (1) | ns. |
| Postprocedural granulation at the tracheostoma n (%) | 1 (1) | 2 (2) | 0 (0) | 0 (0) | 0 (0) | 0 (0) | 1 (1) | 4 (1) | ns. |
| Postprocedural wound healing n (%) | 1 (1) | 0 (0) | 0 (0) | 0 (0) | 0 (0) | 1 (1) | 0 (0) | 2 (0) | ns. |
| Intraprocedural O_2_ desaturation n (%) | 2 (2) | 1 (1) | 0 (0) | 3 (3) | 0 (0) | 4 (4) | 0 (0) | 10 (1) | ns. |
| Intraprocedural hypotension n (%) | 5 (5) | 3 (2) | 5 (11) | 4 (4) | 1 (4) | 7 (6) | 5 (3) | 30 (4) | ns. |
| Intraprocedural cardiac arrhytmia n (%) | 1 (1) | 0 (0) | 0 (0) | 0 (0) | 0 (0) | 0 (0) | 0 (0) | 1 (0) | ns. |
| Intraprocedural fracture of tracheal cartilage n (%) | 1 (1) | 9 (7) | 0 (0) | 7 (7) | 1 (4) | 5 (4) | 6 (4) | 29 (4) | ns. |
| Intraprocedural resuscitation n (%) | 1 (1) | 0 (0) | 0 (0) | 2 (2) | 0 (0) | 2 (2) | 0 (0) | 5 (1) | ns. |
| Intra/Postprocedural death n (%) | 0 (0) | 0 (0) | 0 (0) | 0 (0) | 0 (0) | 0 (0) | 0 (0) | 0 (0) | ns. |

**Table S3.** Procedural related complications during and after PDT (based on seven treatment groups). p-values < 0.05 were considered as significant; ns: non-significant.
